# Proteinuria and hypoalbuminemia are risk factors for thromboembolic events in patients with idiopathic membranous nephropathy: an observational study

**DOI:** 10.1186/1471-2369-13-107

**Published:** 2012-09-10

**Authors:** Sanjeev Kumar, Ananda Chapagain, Dorothea Nitsch, Muhammad M Yaqoob

**Affiliations:** 1 Department of Renal Medicine and Transplantation, Royal London and St Bartholomew’s Hospitals, London, UK; 2 Faculty of Epidemiology and Population Health, London School of Hygiene and Tropical Medicine, London, UK

**Keywords:** Membranous nephropathy, Thromboembolism, Proteinuria, Hypoalbuminemia

## Abstract

**Background:**

Patients with nephrotic syndrome are at an increased risk of thromboembolic events (TEs). However, this association has not been thoroughly investigated in adult patients with idiopathic membranous nephropathy (IMN).

**Methods:**

A retrospective analysis of all 101 consecutive adult patients with MN diagnosed at our centre during 1995 to 2008 was performed. Pertinent data including thromboembolic events and the risk factors for TEs were recorded.

**Results:**

The cohort was followed for 7.2 ± 3 years. Out of 78 patients with IMN, 15 (19.2%) had at least one TE. No TEs occurred six months after diagnosis. The incidence of TEs in the first 6 months of diagnosis was 7.69% (95%CI, 2.5-17.0) and all patients except one had venous TEs. At the time of diagnosis of MN, the patients with TEs had lower serum albumin (1.9 ± 0.5 vs. 2.4 ± 0.4 g/dl, TE vs. no TE; p < 0.01) and greater serum cholesterol (414 ± 124 vs. 317 ± 108 mg/dl, TE vs. no TE; p = 0.01) and 24-h proteinuria (10.7 ± 3 vs. 7.1 ± 4 g, TE vs. no TE; p < 0.01). Multivariate logistic regression adjusted for age, sex, cholesterol and creatinine revealed, an odds ratio of 0.8 (95% CI 0.7 – 0.96; p = 0.01) for every one g/dl increase in baseline serum albumin and, an odds ratio of 1.3 (95% CI 1.05-1.58; p = 0.01) for one gram increase in 24-h proteinuria, for TEs.

**Conclusions:**

Our study finding confirms IMN as a prothrombotic state particularly in the first six months of diagnosis. Proteinuria, in addition to hypoalbuminemia, is a risk factor for TEs. These results have important implications for clinical care of patients with IMN, particularly with regards to initiation and duration of prophylactic anticoagulation.

## Background

Membranous nephropathy (MN) is the most common cause of nephrotic syndrome in Caucasian adults [1]. Patients with nephrotic syndrome are at increased risk of thromboembolic events (TEs) among whom, patients with membranous nephropathy are at an exaggerated risk [2-4]. In 1980s, few case series found that the thromboembolic events occur in up to 20% of adult patients with MN [5-8]. Subsequently, in early 1990s, Bellomo et al. in a letter to the editor reported that a significantly greater proportion of MN patients with thromboembolic events had nephrotic syndrome with serum albumin of less than 25 g/l as compared to patients without thromboembolic events. Based on these published data, it is widely believed that such patients should be prophylactically anticoagulated if serum albumin is less than 20 – 25 g/l [9]. A recent large retrospective study found an eight times higher absolute risk of venous TEs in patients with nephrotic syndrome and the ratio of proteinuria to serum albumin, not serum albumin alone, predicted venous TEs in their cohort of patients with nephrotic syndrome of varied causes [4]. However, the IMN patients were not specifically the focus of investigation.

Investigators have suggested that randomized clinical trials should be conducted to identify whom to administer prophylactic anticoagulation, for how long and in to determine whether anti-coagulation in adult MN patients would improve overall outcomes [10]. However, for trials involving idiopathic MN (IMN) patients key information that may help to design such studies are still lacking, in particular with regards to the timing of thromboembolic events with respect to the diagnosis, their relationship to disease activity and the risk factors. Moreover, the conditions associated with MN (secondary MN) such as lupus [11], human immunodeficiency viral infection [12] or malignancies [13] are themselves pro-thrombotic and *per se* require anti-coagulation irrespective of the presence of nephrotic syndrome. This means that these MN patients, if they have other indications for anticoagulation, may be not eligible to be randomised to anticoagulation.

Though the pathophysiological mechanisms of thromboembolic events in IMN patients are poorly understood, one of the major underlying mechanisms for such complication is the nephrotic syndrome with increased urinary loss of plasma proteins such as plasminogen (component of fibrinolytic system), factors IX, X and XII (components of the coagulation system) and antithrombin III (endogenous anticoagulant) [14-18]. The urinary losses of such plasma proteins correlate with the degree of proteinuria [14,16]. Therefore, we hypothesized that the degree of proteinuria could be a risk factor for thromboembolic events in patients with IMN. If so, this may aid in better risk stratification of patients with IMN with respect to TEs and anti-coagulation. To the best of our knowledge, TEs have not been thoroughly investigated in the adult IMN patients. Therefore, we performed a retrospective analysis of the prospectively collected data on all consecutive biopsy-proven adult IMN patients diagnosed at our centre (i) to characterize the TEs (ii) to determine the timing of the thromboembolic events relative to the diagnosis and (iii) to identify, if any, risk factors for thromboembolic events in such patients.

## Methods

### Study patient characteristics

The prospectively collated computerized data of all consecutive biopsy-proven adult cases of membranous nephropathy diagnosed at our centre between January 1995 and December 2010 were reviewed. Patients who at the time of study entry (January 1995) were ≥ 18 years of age were included. The diagnosis of nephrotic syndrome was confirmed by proteinuria of ≥ 3.5 g/day, derived from a 24-h urine collection. All biopsies were examined by light microscopy and direct immunofluoroscence (for immunoglobulin-G, -M, -A, C3, C1q). Congo red stain was also performed on all the biopsies. Histological diagnosis of membranous nephropathy was made if: (i) normal basement membrane on light microscopy was associated with evidence of IgG deposits on histochemical studies (ii) presence of basement-membrane spikes (iii) incorporation of deposits into the basement membrane or (iv) markedly thickened basement membrane. MN was considered to be secondary if it occurs in the setting of known secondary causes of MN including malignancy, infections, auto-immune disorders and drugs. The development of a secondary illness that is well recognized to be associated with MN (such as malignancy) during the observation period, to suggest a secondary cause of IMN was categorized as secondary MN. Therefore, the patients diagnosed with MN during the last couple of years (2007/8) of the study period were followed up for two more years (2009/10). All patients had age-appropriate screening for causes of secondary MN along with serological tests including hepatitis B, C, HIV, serum complements, serum immunoglobulins, rheumatoid factor, serum protein electrophoresis as well as anti-nuclear antigen (ANA), anti-neutrophilic cytoplasmic antigen (ANCA) and anti-glomerular basement membrane (anti-GBM) antibodies performed which were negative.

Baseline demographics and relevant biochemical data collected included age at presentation, sex, ethnicity, baseline serum creatinine, serum albumin, serum cholesterol and 24 h-urine proteinuria. Glomerular filtration rate was estimated using the abbreviated Modification of Diet in Renal Disease Study equation 186.3 × serum creatinine ^-1.154^ × age ^-0.203^ × 0.742 (if female patient) × 1.212 (if black). MN was considered to be active if the patient had proteinuria of ≥ 3.5 g/day. The medical records were also reviewed for exposure to risk factors for thromboembolic events including major surgery, trauma, malignancy, immobilization for more than 1 week and pregnancy. History of previous thromboembolic events was also recorded.

### Diagnosis of thromboembolic events

The TEs were established by review of the aforementioned prospectively collated computerized case-notes. The patients were considered to have had a thromboembolic event if the event was clinically apparent and confirmed by diagnostic studies. In addition, telephonic interview was also employed to ensure that the patients, who were identified and classified as “no thromboembolic event cohort”, had no TEs during the course of their disease. Clinical evidence of thromboembolic events (arterial or venous) included edematous or painful limbs, dyspnoea, hypoxaemia, chest pain (ischemic or pleuritic), haemoptysis, or other features of deep vein thrombosis or pulmonary embolism. Clinically, cerebrovascular event was considered to occur in the presence of neurological symptoms and signs. Diagnostic confirmation included findings of doppler vascular ultrasonography, ventilation-perfusion scanning (V/Q scan), computed tomography pulmonary angiography (CTPA), and computed tomographic brain scan (CT brain). The diagnosis of a thromboembolic event was confirmed by using radiological criteria as per Royal society of radiology and British thoracic society guidelines. Briefly, consistent non-compressibility of the lower limb venous segment on doppler ultrasound was diagnostic of deep vein thrombosis. V/Q scan results were categorized as high probability for pulmonary thrombosis if there were 1 or more segmental perfusion defect(s) with normal ventilation or 2 or more large subsegmental perfusion defects (>75% of a segment) with normal ventilation. The finding of an intraluminal filling defect within a pulmonary arterial vessel was considered to be confirmatory of pulmonary embolism.

### Statistical analysis

Results are expressed as mean ± standard deviation (sd) for continuous data and as percentages for categorical data. The significance of differences in clinical and laboratory data between the groups was tested by unpaired t–test, Mann–Whitney *U*-test, Wilcoxon signed-rank test, and chi-square test as appropriate. To identify risk factors for thromboembolic events, univariable and multivariable forward logistic regression analysis was performed (SPSS software version 12). The inclusion criterion for a variable was based on the score statistic i.e. the variable with the most significant score statistic was added to the model. Likelihood ratio statistic and Wald criterion was employed to exclude the variable. Complete data sets for multivariable analysis were available in 52 out of 62 patients (84%) in the “no TE” group and 14 out of 15 (93%) in TE group. An exact 95% confidence interval was calculated for the 6 months incidence rate in patients who did not have a TE before or at the time of diagnosis. All statistical analyses were two sided, and *P* < values less than 0.05 were considered statistical significance.

## Results

### Study patients

**Figure 1:**
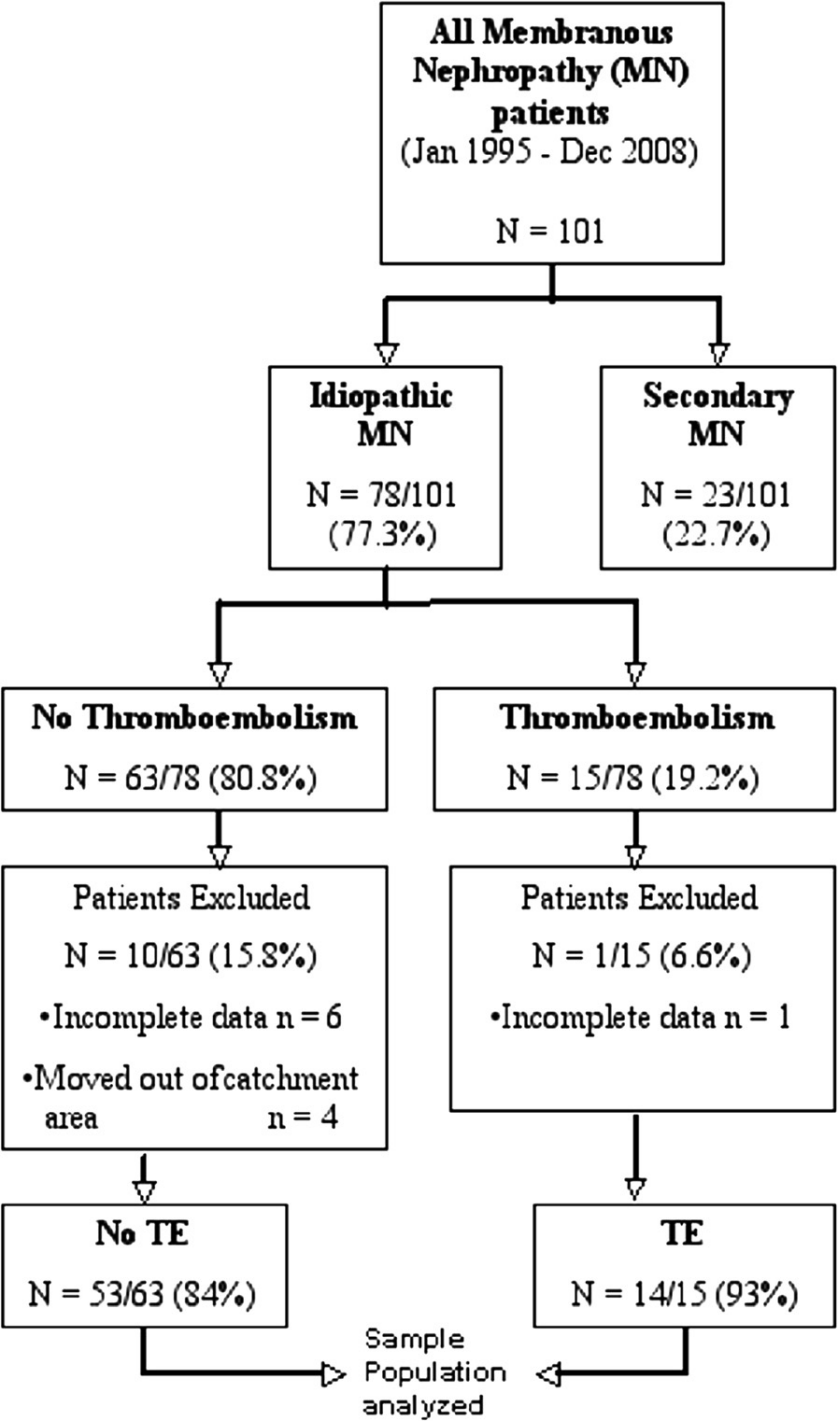
Flow diagram showing all consecutive biopsy-proven membranous nephropathy patients diagnosed at our centre between January 1995 and December 2008.

Between 1^st^ January 1995 and 31^st^ December 2008, a total of 101 patients were diagnosed to have biopsy-proven membranous nephropathy at our centre (Figure 1). Twenty-three out of 101 patients (22.7%) were considered to have secondary MN. The remaining 78 (77.3%) were diagnosed to have idiopathic MN and constituted the study population. Secondary causes of MN were malignancies (lymphoma, n = 3; solid organ, n = 3); infections (hepatitis-B, n = 3; human immunodeficiency virus, n = 2); auto-immune disorders (systemic lupus erythematosus, n = 9; systemic sclerosis, n = 1; anti-phospholipid syndrome, n = 1) and drugs (pencillamine therapy, n = 1).

Among the 78 patients with IMN, 15 (19.2%) had at least one episode of thromboembolic events (TE group); the remaining 63 patients (80.8%) had no episode of thromboembolic events (No TE group). The average age of our entire MN cohort was 58 ± 16 years with 42 out of 66 (63%) being male. The patients were from varied ethnic backgrounds including Caucasians (59%), Afro-Carribeans (16%), South-Asians (13%) and Middle-East (12%). The cohort was followed for 7.2 ± 3 years. The mean baseline eGFR was 65 ± 27 (ml/min per 1.73 m^2^) and mean serum albumin and cholesterol was 2.3 ± 0.4 g/dl and 336 ± 128 mg/dl, respectively. The average 24-h proteinuria was 7.9 ± 3.5 g.

### Treatment

Patients were commenced on immuno-supressive therapy, if they continued to remain nephrotic in spite of three months of maximal medical treatment as per our unit’s policy. In addition, warfarin anti-coagulation prophylaxis was commenced at the same time if the serum albumin remained persistently below 25 g/dl. Out of 78 patients with primary membranous nephropathy, 49 patients were immunosupressed on the treating clinician’s discretion. Thirty-two (66.6%) patients were induced in remission with oral cyclophosphamide and prednisolone. Six with prednisolone alone, six patients were induced with prednisolone and azathioprine, two with prednisolone and mycophenolate mofetil and a further three with calcineurin inhibitors (two with ciclosporin and one with tacrolimus).

### Thromboembolic events

Among 78 IMN patients, fifteen patients (19.2%) suffered TE. Data was not available on one patient with TE and hence, was excluded from further analysis (Figure 1). The median age at presentation with a TE was 59 years (range 22-82 yrs) and 11 out of 14 (78%) patients with TE were male. The most commonly encountered TE was pulmonary embolism (PE, n = 9, 64%) followed by lower extremity deep vein thromboses (DVT, n = 3, 21%). One patient had arterial thromboembolic event in the form of a CVA. All patients with PE presented with shortness of breath and were noted to be hypoxic except patient 13 (Table 1.) who presented with haemoptysis. The patients with DVT presented with asymmetrical swelling of calves, in addition, to pedal oedema. One patient had renal vein thrombosis (RVT) and presented with bilateral loin pain and pedal oedema. The patient with CVA presented with left hemiparesis. The latter patient (Table 1), a non-smoker, previously fit and well, was diagnosed with IMN three months prior to the onset of neurological symptoms. The diagnosis of IMN was established on the basis of bilateral pedal edema (four month duration), serum albumin, 2.1 g/dl, serum cholesterol, 440 mg/dL, 24 h-proteinuria, 10.4 g/24 h and renal biopsy findings of MN. Whilst under close follow-up, he suffered from CVA and the CT scan brain revealed a lacunar infarct in the right middle cerebral artery territory. The other battery of investigations including blood glucose, thrombophilia screen, auto-immune profile, CT angiogram of cranial vessels, carotid dopplers and echocardiography were all unremarkable. Hence, the thrombotic event was attributed to the nephrotic syndrome.

**Table 1:** Patients with idiopathic membranous nephropathy with thromboembolic events*

	**Age (y)**	**Sex**	**Year of diagnosis**	**Type of event, test (treatment)**	**Serum albumin (mg/dl), proteinuria (g/24 h)**	**Time to diagnosis (mth)**
1	68	M	1995	PE, V/Q (Pred + CycloP)	1.8, 14	- 2
2	43	M	1998	PE, V/Q (Pred + CycloP)	1.7, 14	- 1
3	82	M	1999	IleofemDVT^#^, US (spontaneous remission)	2.0, 12	+ 6
4	58	M	2000	PE, V/Q (Pred + CycloP)	1.9, 11	0
5	58	F	2001	RVT, US (Pred + CycloP)	2.1, 7	0
6	68	F	2002	PE, CTPA (Pred + CycloP)	2.2, 5	0
7	79	M	2002	PE^#^, CTPA (Pred + MMF)	2.8, 9	+ 2
8	72	M	2003	CVA^#^ (Pred + MMF)	1.7, 14	+ 3
9	50	M	2003	DVT, US (Pred + CycloP)	1.5, 12	- 2
10	64	F	2004	DVT, US (Pred + CycloP)	2.2, 5	- 0.5
11	42	M	2005	PE, CTPA (spontaneous remission)	2.0, 12	0
12	22	M	2005	PE, CTPA (Pred + CsA)	1.2, 12	0
13	39	M	2005	PE^#^, CTPA (Pred + CycloP)	1.9, 11	+ 1
14	61	M	2007	PE^#^, CTPA (Pred + CycloP)	1.6, 12	+ 1

*14/15 had complete data sets.

*PE*, pulmonary embolism; *V/Q*, ventilation/perfusion; *RVT*, renal vein thrombosis; *CTPA*, computed tomographic pulmonary angiogram; *CVA*, cerebrovascular accident; *DVT*, deep vein thrombosis; *Pred*, prednisolone; *CsA*, ciclosporin; *CycloP*, cyclophosphamide; *MMF*, mycophenolate mofetil.

All the patients who presented with TEs (patient 1, 2, 4, 5, 6, 9 – 12; 64%) were immediately anticoagulated on confirmation of the diagnosis of TE.

^**#**^Not on prophylactic anti-coagulation.

We next looked at the timing of the diagnosis of TEs and IMN. Nine out of fourteen patients (64%) presented with a thromboembolic event and nephrotic-range proteinuria that led to the diagnosis of IMN. Out of these nine patients, five (36%) were immediately diagnosed, whereas four (28%) patients had TEs 2, 1, 2 and 0.5 months *before* the diagnosis was established. The latter group represents the cohort where the diagnosis of nephrotic syndrome was not suspected and hence the delay in diagnosis. Among the patients whose diagnosis was delayed by more than 15 days, two of them suffered from pulmonary embolism and the third patient (Table 1.) had deep vein thrombosis. The first two patients with PE, presented to the accident and emergency department via their general practitioners (GPs) with 1 – 2 week history of intermittent shortness of breath and pleuritic chest pain. The third patient with DVT presented with a 10-day history of bilateral swollen calves via the similar route. Past medical history was unremarkable for all three patients. On presentation, they were haemodynamically stable with unremarkable chest X-ray. An emergency V/Q scan confirmed the diagnosis of PE (Table 1, patient 1 and 2) and the diagnosis of DVT was confirmed in the third patient via an immediate lower limbs doppler ultrasound scan. The possibility of nephrotic syndrome as the underlying etiology was not entertained and the patients were referred back to their GPs on anti-coagulants. The persistence of symptoms led to repeat laboratory investigations that included serum albumin. Further investigations including urine dipstick analysis revealed the diagnosis. The remaining five patients suffered VTE within six months after the diagnosis of IMN yielding an incidence rate of 7.69% (95% confidence interval, 2.54% - 17.05%) over 6 months. These patients were not on any prophylactic anti-coagulation. We reviewed all patients at the end of the study period and we could not identify any patients in our cohort with IMN who went on to develop VTEs after six months of diagnosis of IMN whilst they were under regular follow-up. All the patients had active MN when they presented with thromboembolic event. All the patients who presented with TEs were immediately anticoagulated on confirmation of the diagnosis of TE.

### Risk factors

Baseline characteristics and clinical data of patients with idiopathic membranous nephropathy at the time of renal biopsy i.e. at the time of diagnosis, is summarized on Table 2. None of the patients in the TE group had trauma, surgery, or previous history of thromboembolic events or immobilization. The two groups (TE vs. no TE) were similar with respect to age (57 ± 17 vs 58 ± 16 years), sex (males, 78% vs. 73%), ethnicity (Afro-Carribeans, 14% vs. 17%), duration of follow-up (7.8 ± 3 vs. 7.1 ± 3 years) and renal function (eGFR, 62 ± 29 vs. 66 ± 25 ml/min per 1.73 m^2^). Compared with IMN patients who did not have an event, those who had a TE had significantly greater 24 h-proteinuria (10.7 ± 3 vs. 7.1 ± 4 g, TE vs. no TE; p < 0.01), hypoalbuminemia (1.9 ± 0.5 vs. 2.4 ± 0.4 g/dl, TE vs. no TE; p < 0.01) and higher serum cholesterol (414 ± 124 vs. 317 ± 108 mg/dl, TE vs. no TE; p < 0.05). On multivariable analysis, serum albumin (odds ratio, 0.8; 95% confidence interval, CI: 0.7 – 0.96, p = 0.01) and 24 h-proteinuria (odds ratio, 1.3; 95% CI: 1.05 – 1.58, p = 0.01) were found to be independent predictors of TEs (Table 3). A test of full model against a constant only model was statistically significant, indicating predictors as a set reliably distinguished between patients with TEs and no TEs (chi square = 16.98, p < 0.000 with degrees of freedom = 2).

**Table 2:** Baseline characteristics and clinical data of patients with idiopathic membranous nephropathy at the time of renal biopsy*

**Characteristic**	IMN patients*(n = 67)	**No Thromboembolic events (n = 53)**	**Thromboembolic events (n = 14)**	***P*-value**
Age (years)	58.3 ± 16.7	58.6 ± 16.7	57.3 ± 17.4	*NS*
Sex, male,n (%)	42 (63%)	38 (73%)	11 (78.5%)	*NS*
Ethnicity, n (%)				
Caucasians	39 (59%)	31 (60%)	8 (58%)	*NS*
Afro-Carribeans	11 (16%)	09 (17%)	2 (14%)	*NS*
South Asians	09 (13%)	07 (14%)	2 (14%)	*NS*
Middle-East	08 (12%)	05 (09%)	2 (14%)	*NS*
Duration of follow-up (years)	7.2 ± 3	7.1 ± 3	7.8 ± 3.2	*NS*
s.creatinine (mg/dl)	1.24 ± 0.8	1.23 ± 0.7	1.28 ± 0.8	*NS*
s.albumin (g/dl)	2.3 ± 0.4	2.4 ± 0.4	1.9 ± 0.5	< 0.01
s.cholesterol (mg/dl)	336 ± 128	317 ± 108	414 ± 124	0.01
eGFR (ml/min per 1.73 m^2^)	65 ± 27	66 ± 25	62 ± 29	*NS*
Proteinuria (g/24 h)	7.9 ± 3.5	7.1 ± 4	10.7 ± 3	<0.01
Immuno-suppresion	49 (73.1%)	35 (66%)	12 (86%)	

* IMN patients with complete data (n = 67/78, 85.8%): IMN patients with no TE and with TE, 84% (n = 53/63) and 93% (n = 14/15); respectively.

**Table 3:** Adjusted^*^Odds Ratio for Risk of Thromboembolic Events in patients with idiopathic membranous nephropathy

**Variable**	**Odds Ratio (95% CI)**	***P*- value**
s.albumin (per g/dl)	0.8 (0.7 – 0.96)	0.01
24 h-proteinuria (per g/24 h)	1.3 (1.05 - 1.58)	0.01
Male	0.7 – 2.1	*NS*
s.cholesterol	0.86 – 1.5	*NS*
s.creatinine	0.79 – 3	*NS*

* Adjusted for age, sex, serum cholesterol and serum creatinine.

Because no TEs occurred after 6 months of diagnosis of IMN, we next looked at proteinuria and serum albumin as markers of disease activity at 6 months. Overall, all patients had either partially or completely remitted, with none of the patients left with nephrotic range proteinuria. There was no significant difference between the mean serum abumin (3.72 ± 0.06 g/L vs. 3.65 ± 0.08 g/L; no TE vs. TE group; respectively) nor 24 h mean proteinuria (0.95 ± 0.17 g vs. 1.09 ± 0.29 g; no TE vs. TE group; respectively) at 6 months between the two groups. Two thromboembolic events occurred in the secondary MN group both of which were associated with systemic lupus erythematosus.

## Discussion

To the best of our knowledge, this is the first study that describes the timing of the TEs relative to the diagnosis and (ii) the risk factors (including disease activity) for TEs in patients with idiopathic membranous nephropathy. We found that the majority of TE events occurred before or at diagnosis of IMN and 7.4% suffered a TE over the next 6 months after the IMN diagnosis was established. No events occurred after 6 months of IMN diagnosis in our study population. Moreover, in patients with TE events compared to those without TE events, proteinuria and serum albumin are found to be independent risk factors for thromboembolic events in adult IMN patients.

In our study, 19% of IMN patients (when analysed cross-sectionally) had at one time in their disease course a TE (either prior to or directly after IMN diagnosis). Our finding suggests that the incidence rate of first thromboembolic event in IMN patients is high when compared to the available rates in general population (0.3 per 100 person-years) and in patients with lupus (1 per 100 person-years) [11,19]. It is comparable to patients with Wegener’s granulomatosis (7 per 100 person-years) and people who had a previous deep vein thrombosis (7 per 100 person-years) [20,21]. The incidence of TE in our cohort (7.6% over 6 months) is less than the incidence reported by Bellomo and colleagues in their MN cohort (16.9%). This could be partly explained by the difference in the proportion of immunosuppressed patients to treat the nephrotic range proteinuria (25% of their patients with TE were immunosuppressed versus 100% of our patients with TE). However, how the incidence was measured was not mentioned in the latter study. A recent retrospective cohort study confirmed high absolute risk (approximately eight times) of venous TEs in patients with nephrotic range proteinuria and in addition, found a high annual incidence of 1.40 among the MN patients with nephrotic syndrome [4]. However, the study was not specifically aimed at studying MN patient and included patients with as diverse as patients with diabetic nephropathy (28%) and nephrotic syndrome not otherwise specified (46%). In both these studies, no information was available on the underlying etiology of MN (i.e. primary vs secondary), the relation of TEs to the timing of diagnosis and disease activity and the predictors specific to IMN patients.

Another question is why IMN patients are at an exaggerated risk of TEs when compared to patients with other causes of similar profound non-selective nephrotic range proteinuria such as FSGS or amyloid nephropathy. It is tempting to hypothesize that perhaps the underling trigger unique to IMN pathology render IMN patients at higher risk of TEs. We found that all the TEs occurred when the IMN was active. This suggests that the exaggerated risk of TEs in IMN patients is due to the underlying nephrotic syndrome.

In our study, pulmonary embolism was the most common thromboembolic event followed by deep vein thrombosis. Our findings are in line with those reported by Bellomo et al. A recent review of the coding data from the discharged patients in the United States with nephrotic syndrome found deep vein thrombosis in 1.5% and PE in 0.5% and less than 0.5% had renal vein thrombosis [22]. In contrast, the studies conducted in late 1970s and early 1980s reported renal vein thrombosis as the commonly encountered TE in MN patients [5-7,14]. This discrepancy may be explained by the fact that the latter studies evaluated patients with nephrotic syndrome with renal venography to specifically determine renal vein thrombosis.

Bellomo et al. found that the baseline serum albumin and 24-h proteinuria, between the patients who suffered a TE and those with no TE, were similar, though significantly greater proportion of patients in the TE group had nephrotic syndrome with albumin < 2.5 g/l. Based on this observation, it was concluded that the presence of the nephrotic syndrome and of a low serum albumin (< 2.5 g/l) at presentation was associated with a significantly greater risk of TE. Our study, for the first time, has formally quantified the increased odds of TE associated with lower serum albumin in adult IMN patients. In addition, we also find that the magnitude of proteinuria should be taken into account while assessing the risk of TE in patients with IMN. The 24-h proteinuria greater than 10 g/day could be regarded as an independent risk factor for thromboembolic events in patients with IMN, irrespective of the serum albumin. This is not surprising as one of the major mechanisms of thrombophilia in IMN patients is urinary loss of critical proteins involved in coagulation including plasminogen, factors IX, X and XII, and antithrombin III [14-16]. None of our patients after 6 months of diagnosis had thromboembolic events. One may therefore consider primary thromboprophylaxis in the first six months following the diagnosis of IMN especially in patients with significant proteinuria (>10 g/day) and/or serum albumin (<20 g/dl). Similar to our finding, the Midwest Pediatric Nephrology Consortium Study (MWPNC study), found proteinuria (*p* < 0.0001) as a significant independent risk factor of TE in children with nephrotic syndrome [23]. The possible link between microalbuminuria or proteinuria and the risk of thromboembolic events have also been demonstrated in the general population, in patients with atrial fibrillation and in a cohort of hospitalized patients with nephrotic syndrome who underwent imaging study for TE [22,24-26].

Our study has all limitations that are inherent to observational studies. These include confounding by other factors, which we have not measured. We had clear diagnostic criteria so exposure or outcome misclassification is unlikely. Our study lacks the detailed thrombophilia profile of the study population. In spite of the above limitations, these results may have important implications for clinical care of patients with IMN. Our study provides vital information that may help in, firstly, regarding the timing and the possible duration of prophylactic anti-coagulation; secondly, about the patient number that could benefit from prophylactic anti-coagulation; and thirdly, identification of proteinuria, as an independent risk factor that may aid in better risk assessment of such patients. In addition, the demographic characteristics of our patient population, including patients from various ethnicities, support the generalizability of our findings to IMN patients elsewhere. The results of our study may help in appropriate power calculation of future prospective randomized controlled trials in IMN patients with regards to thromboprophylaxis. Furthermore, the findings highlight the importance of urine dipstick testing in patients with TE, as TE could be a presenting clinical feature of nephrotic syndrome, in particular membranous nephropathy. Further prospective large studies are required to confirm the findings of our study.

## Conclusions

The severity of proteinuria, in addition to hypoalbuminemia, should be taken into account while evaluating the risk for thromboembolic events in patients with IMN. The risk is greatest in the first six months of diagnosis. Urine dipstick analysis should be performed as a part of initial evaluation of all patients presenting with thromboembolic events. These results have important implications for clinical care of patients with IMN, particularly with regards to initiation and duration of prophylactic anticoagulation.

## Competing interest

The authors declare that they have no competing interests.

## Authors’ contributions

SK and MMY conceived the study, participated in its design and coordination and helped to draft the manuscript. AC participated in its design and coordination and helped to draft the manuscript. DN performed the statistical analysis. All authors read and approved the final manuscript.

## Pre-publication history

The pre-publication history for this paper can be accessed here:

http://www.biomedcentral.com/1471-2369/13/107/prepub
